# Soluble CD206 levels correlate with disease deterioration and predict prognosis of anti‐MDA5 antibody‐positive dermatomyositis related interstitial lung disease

**DOI:** 10.1111/crj.13616

**Published:** 2023-04-11

**Authors:** Yan Li, Xiaoqin Liu, Mi Tian, Ruyi Zou, Yujuan Gao, Mei Huang, Kefeng Zhou, Min Cao, Hourong Cai

**Affiliations:** ^1^ Department of Respiratory Medicine The Affiliated Drum Tower Hospital of Nanjing University Medical School Nanjing Jiangsu China; ^2^ Department of Radiology The Affiliated Drum Tower Hospital of Nanjing University Medical School Nanjing Jiangsu China

**Keywords:** CD206, clinically amyopathic dermatomyositis, dermatomyositis, interstitial lung disease, MDA5

## Abstract

**Introduction:**

The prognosis of anti‐MDA5 antibody‐positive dermatomyositis/clinically amyopathic dermatomyositis‐associated interstitial lung disease (MDA5‐DM/CADM‐ILD) is poor. This study was to evaluate the effect of serum soluble CD206 (sCD206), a biomarker of macrophage activation, on predicting the interstitial lung disease (ILD) deterioration and prognosis for MDA5‐DM/CADM‐ILD.

**Methods:**

Forty‐one patients diagnosed with MDA5‐DM/CADM‐ILD were retrospectively included. The clinical data were analyzed. Serum sCD206 levels were measured in 41 patients and 30 healthy controls. The relation between sCD206 levels and ILD deterioration was assessed. Receiver operating characteristic (ROC) curve was generated to determine the optimal cut‐off value of sCD206 for predicting outcome. The association between sCD206 and survival was examined.

**Results:**

The median serum sCD206 level in patients was significantly higher than healthy controls (464.1 ng/mL vs. 349.1 ng/mL, *P* = 0.002). In DM/CADM patients, the sCD206 level was significantly higher in patients with acute/subacute interstitial lung disease (AILD/SILD) than those with chronic interstitial lung disease (CILD) (539.2 ng/mL vs. 309.4 ng/mL, *P* = 0.005). The AUC of sCD206 was 0.885 for predicting mortality (95% CI 0.779–0.990). Patients were divided into two groups: sCD206 high level group (≥400 ng/mL) and sCD206 low level group (<400 ng/mL). Patients with sCD206 high level had significantly decreased survival rate than those with low level (25% vs. 88%, *P* < 0.001). The adjusted hazard ratio of sCD206 for mortality was 1.003 (adjusted for age and gender, *P* < 0.001), with sCD206 high level associated with higher death risk (HR 4.857, *P* = 0.006).

**Conclusion:**

Serum sCD206 might be a potential predictor of ILD deterioration and prognosis for Chinese patients with MDA5‐DM/CADM‐ILD.

## Graphical abstract

Serum soluble CD206 (sCD206) was elevated in MDA5‐DM/CADM‐ILD patients with acute/subacute interstitial lung disease (AILD/SILD) compared with chronic interstitial lung disease (CILD). Serum sCD206 high level was associated with shorter survival in MDA5‐DM/CADM‐ILD patients.

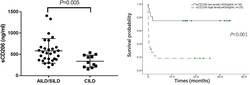

## 1 INTRODUCTION

Dermatomyositis (DM) is an idiopathic inflammatory myopathies (IIM) affecting multiple organs including skin, muscles, and lungs.^1^ Clinically amyopathic dermatomyositis (CADM) is a unique subtype of DM presenting with typical rash but without muscular manifestations. DM often affects the lungs and presents with interstitial lung disease (ILD), especially in patients with CADM.^2^ ILD is the leading cause of death from DM/CADM.^3^, ^4^ Notably DM/CADM patients with antimelanoma differentiation‐associated gene 5 (anti‐MDA5) antibody positive frequently develop a rapidly progressive form of ILD with fatal outcome.^5^ Especially those patients presenting with active disease pattern may die of respiratory failure in very short term and need to be identified timely in order to take better clinical management. Serological markers can be measured noninvasively, even in patients severely ill. Useful serum biomarkers would be helpful to evaluate the ILD deterioration and predict prognosis for patients with anti‐MDA5 positive DM/CADM‐related ILD (MDA5‐DM/CADM‐ILD).

Macrophages involving in the pathogenesis of DM/CADM‐related ILD (DM/CADM‐ILD) has received increasing interest during recent years. There are two types of macrophages: M1 type (classically activated) and M2 type (alternatively activated).^6^ M1 macrophages have proinflammatory functions, whereas M2 macrophages show anti‐inflammatory characteristics.^7^ The macrophage mannose receptor (CD206) is a 180‐kDa transmembrane glycoprotein and mainly expressed by M2 macrophages. CD206 has been considered a biomarker of activated M2 macrophages. CD206 can be cleaved from macrophage membrane and released into periphery circulation, called soluble CD206 (sCD206). Similar to membrane form, sCD206 has the function of altering the innate and adaptive immune responses.^8^ Serum sCD206 has been highlighted as a noninvasive biomarker in infectious diseases.^9^, ^10^ However, the value of sCD206 in MDA5‐DM/CADM‐ILD has not been fully examined.

This retrospective study was conducted to evaluate the effect of sCD206 on predicting ILD deterioration and prognosis for MDA5‐DM/CADM‐ILD patients. Meanwhile, the clinical features were assessed to better understand this unique disease.

## 2 MATERIALS AND METHODS

### 2.1 Subjects

Patients with newly diagnosed DM/CADM‐ILD at the Department of Respiratory Medicine of Nanjing Drum Tower Hospital between 2016 and 2018 were retrospectively reviewed. Bohan and Peter criteria were used to make the diagnosis of DM.^1^ Patients who met the definite or probable DM criteria were included. The diagnosis of CADM referred to the Euwer and Sontheimer criteria.^11^, ^12^ The diagnosis of ILD was based on the presence of symptoms such as cough and exertional dyspnea, and requisite high‐resolution computed tomography (HRCT) abnormalities. Only DM/CADM‐ILD patients with anti‐MDA5 antibody positive were recruited. Healthy controls were chosen from health screening conducted at Nanjing Drum Tower Hospital within the same time period between 2016 and 2018. A total of 41 MDA5‐DM/CADM‐ILD cases and 30 healthy controls were identified in the current study. The study adheres to the amended Declaration of Helsinki criteria. The research protocol was approved by the Ethics Committee of Nanjing Drum Tower Hospital. Regarding serum conservation, written informed consent was obtained from each subject. Regarding the data analyses, informed consent was waived because this study was analyzed retrospectively using anonymized clinical data.

### 2.2 Data collection

Full medical record abstraction for each patient was conducted and the following baseline clinical variables were collected: Age, gender, smoking status, DM/CADM diagnosis, clinical symptoms, chest HRCT manifestations, treatment modalities, available pulmonary function test results, laboratory results, and other medical conditions.

### 2.3 Evaluation of ILD deterioration

Regarding ILD deterioration, patients were divided into two groups: Acute/subacute interstitial lung disease (AILD/SILD) and chronic interstitial lung disease (CILD). Patients presented with rapidly progressive ILD and dyspnea within 3 months from onset of respiratory symptoms were grouped as AILD/SILD. Patients presented with stable or slowly progressive ILD and dyspnea more than 3 months from the onset of respiratory symptoms were grouped as CILD.^13^, ^14^ The evaluation of ILD deterioration was made blindly without knowledge of the sCD206 levels.

### 2.4 Classification of HRCT manifestations

Chest HRCT images at the time of diagnosis from all included patients were reviewed. HRCT manifestations were categorized through direct interpretation and analysis by an experienced ILD specialist (HR Cai) and a thoracic radiologist (KF Zhou). According to the guidelines for interstitial pneumonias,^15^ patients' HRCT manifestations were classified as following four types: Organizing pneumonia (OP), nonspecific interstitial pneumonia (NSIP), OP combined with NSIP, and diffuse alveolar damage (DAD). The disagreements were resolved by consensus agreement.

### 2.5 Detection of serum anti‐MDA5 antibody and sCD206

Anti‐MDA5 antibody and sCD206 were detected using the stored serum samples collected at the diagnosis. Anti‐MDA5 antibody was tested by anti‐myositis antibody profile IgG detection kit (EUROMIMMUN Medizinische Labordiagnostika AG, Germany).^16^ Serum sCD206 levels were measured by ELISA (Raybiotech Human CD206 ELISA Kit). Experimental procedures were performed according to the manufacturer's instructions.

### 2.6 Statistical analyses

Data are presented as medians (observed ranges) or numbers (percentages). For continuous variables, Mann–Whitney *U* test was used. For categorical variables, either chi‐square test or Fisher's exact test was used according to the sample size. Spearman's rank correlation was used to evaluate the correlations between sCD206 and other continuous variables. Receiver‐operating characteristic (ROC) curve was generated to determine the optimal cut‐off value of serum sCD206 for predicting patients' outcome. The point that had the highest value of Youden index (sensitivity + specificity − 1) was selected as the optimal cut‐off value.

Survival status was identified by reviewing the medical records or telephone follow‐ups. Overall survival (OS) time was calculated as the period (months) from DM/CADM‐ILD diagnosis (the date of blood draw) to death. Survival was assessed up to December 30, 2018. Patients still alive on December 30, 2018, were censored. Univariate and multivariate Cox hazards models were used to evaluate the prognostic impact on survival for clinical variables. The Kaplan–Meier method with the log‐rank test was used to compare the survival difference between patient groups.

Analyses in this study were performed with SPSS (version 22.0, SPSS Inc., Chicago, IL, USA) and GraphPad Prism (Version 7, GraphPad Software, San Diego, CA, USA). The *P* values were two sided and at a significance level of 0.05.

## 3 RESULTS

### 3.1 Baseline characteristics

**FIGURE 1:**
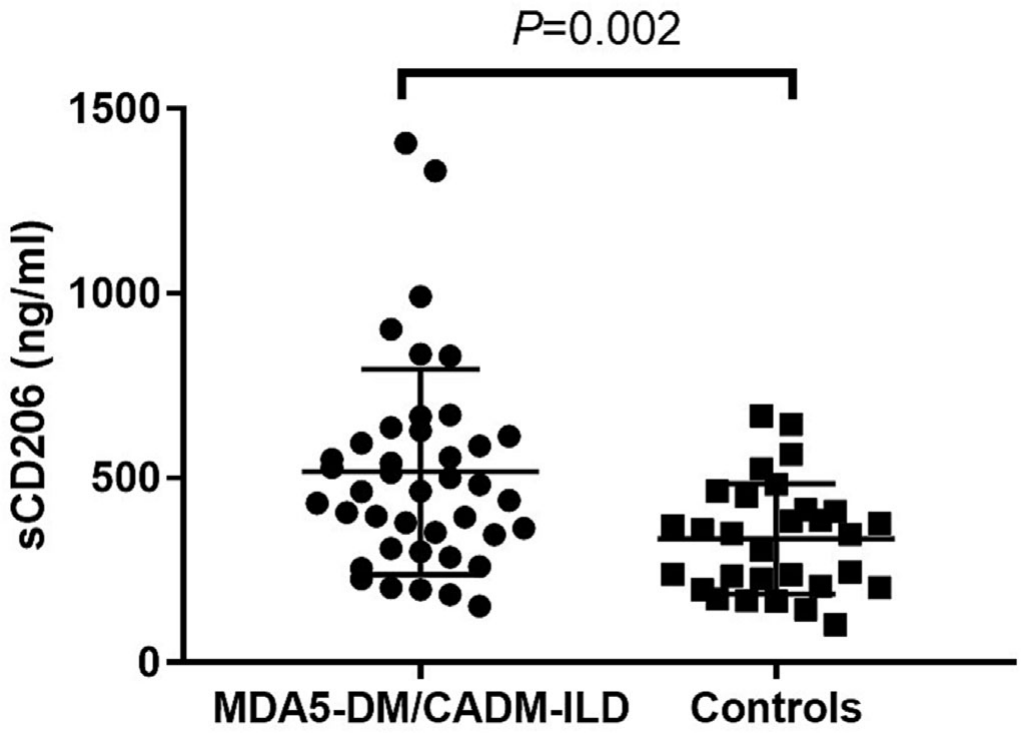
The serum soluble CD206 (sCD206) level was higher in patients with anti‐MDA5 positive dermatomyositis/clinically amyopathic dermatomyositis‐related interstitial lung disease (MDA5‐DM/CADM‐ILD) (*n* = 41) than healthy controls (*n* = 30).

**FIGURE 2:**
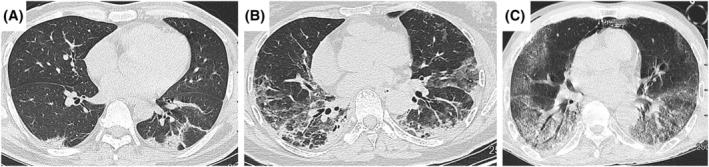
Representative chest HRCT patterns. (A) OP pattern, patchy consolidation and irregular cord opacities distributed predominantly around the basal and peripheral lungs. (B) NSIP pattern, ground glass opacities on both lungs, with traction bronchiectasis. (C) DAD pattern, ground glass opacities diffusely distributed under the pleura of both lungs, with traction bronchiectasis.

Serum sCD206 level was significantly elevated in MDA5‐DM/CADM‐ILD patients compared with controls (median level: 464.1 ng/mL vs. 349.1 ng/mL, *P* = 0.002; Figure 1). Out of 41 patients, 26 died of respiratory failure. The patients' baseline characteristics are presented in Table 1. There was a higher ratio of CADM (58.5%). Thirty patients (73.2%) presented with AILD/SILD. Regarding chest HRCT manifestations, 34.1% (14/41) showed DAD, 29.3% (12/41) showed NSIP, 26.8% (11/41) showed OP combined with NSIP, and 9.8% (4/41) showed OP. The representative chest HRCT images were shown in Figure 2.

**TABLE 1:** Baseline characteristics and the correlations with serum soluble CD206 levels in 41 patients.

Variables	Patients (*n* = 41)	*r*	*P* value
Age, years	53.0 (32.0–77.0)	0.251	0.113
Female	24 (58.5)	‐	
Never/former/current smokers	28 (68.3)/6 (14.6)/7 (17.1)	‐	
DM/CADM	17 (41.5)/24 (58.5)	‐	
AILD/SILD/CILD	12(29.3)/18 (43.9)/11(26.8)	‐	
Fever	21 (51.2)	‐	
HRCT findings		‐	
OP/NSIP/OP + NSIP/DAD	4(9.8)/12(29.3)/11(26.8)/14(34.1)	‐	
Treatment prior to admission		‐	
CS alone/CS + IM/None	18(43.9)/4(9.8)/19(46.3)		
Treatment after admission		‐	
CS alone/CS + IM	5(12.2)/36(87.8)		
FVC, % predicted (*n* = 17)	66.09 (36.1–86.2)	0.230	0.374
FEV1, % predicted (*n* = 17)	69.6 (41.9–95.2)	0.252	0.328
TLC, % predicted (*n* = 11)	71.4 (39.2–84.8)	−0.155	0.650
DLCO, % predicted (*n* = 14)	58.45 (8.2–81.7)	−0.156	0.594
PaO_2_, torr (*n* = 37)	60.0 (44.0–97.9)	−0.399	**0.014**
OI (*n* = 37)	258 (56–710)	−0.490	**0.002**
WBC, 10^9^/L (*n* = 41)	6.9 (1.9–18.0)	0.015	0.924
PLT, 10^9^/L (*n* = 41)	198 (97–347)	−0.210	0.189
Lymphocyte count, /mm^3^ (*n* = 41)	700 (200–2500)	−0.484	**0.001**
CD4+ lymphocyte count, /mm^3^ (*n* = 36)	255.5 (37–1169)	−0.392	**0.018**
NK count, /mm^3^ (*n* = 36)	67.5 (13–1060)	−0.277	0.103
CRP, mg/L (*n* = 38)	11.15 (2.2–79.1)	0.233	0.158
ESR, mm/h(*n* = 33)	40 (7–97)	−0.009	0.961
CK, U/L (*n* = 37)	41.0 (11–377)	0.264	0.115
LDH, U/L (*n* = 38)	402 (227–15 743)	0.602	**<0.001**
Ferritin, ng/mL (*n* = 41)	1143.9 (38.8–27130.0)	0.428	**0.005**
sCD206, ng/mL (*n* = 41)	464.1 (152.5–1407.2)	‐	

*Note*: Data are presented as median (observed range) or number (%). All *P* values are derived from Spearman's rank correlation coefficient. Bold values: *P*<0.05.

Abbreviations: AILD/SILD, acute/subacute interstitial lung disease; CADM, clinically amyopathic dermatomyositis; CILD, chronic interstitial lung disease; CK, creatine kinase; CRP, C‐reactive protein; CS, corticosteroids; DAD, diffuse alveolar damage; DLCO, diffusing capacity of the lung carbon monoxide; DM, dermatomyositis; ESR, erythrocyte sedimentation rate; FEV1, forced expiratory volume in 1 s; FVC, forced vital capacity; HRCT, high‐resolution computed tomography; IM, immunosuppressants; LDH, lactate dehydrogenase; NK, natural killer; NSIP, nonspecific interstitial pneumonia; OI, oxygen index; OP, organizing pneumonia; PLT, platelet; TLC, total lung capacity; WBC, white blood count.

### 3.2 Correlations between serum sCD206 levels and clinical characteristics

Correlation analyses showed that serum sCD206 levels were positively correlated with conventional serum markers for disease activity, such as LDH and ferritin (*P*s < 0.05), while negatively correlated with PaO_2_, oxygen index (OI), peripheral lymphocyte count, and CD4+ lymphocyte count (*P*s < 0.05). The correlation coefficients and *P* values are listed in Table 1.

### 3.3 Serum sCD206 level was elevated in MDA5‐DM/CADM patients with AILD/SILD

**FIGURE 3:**
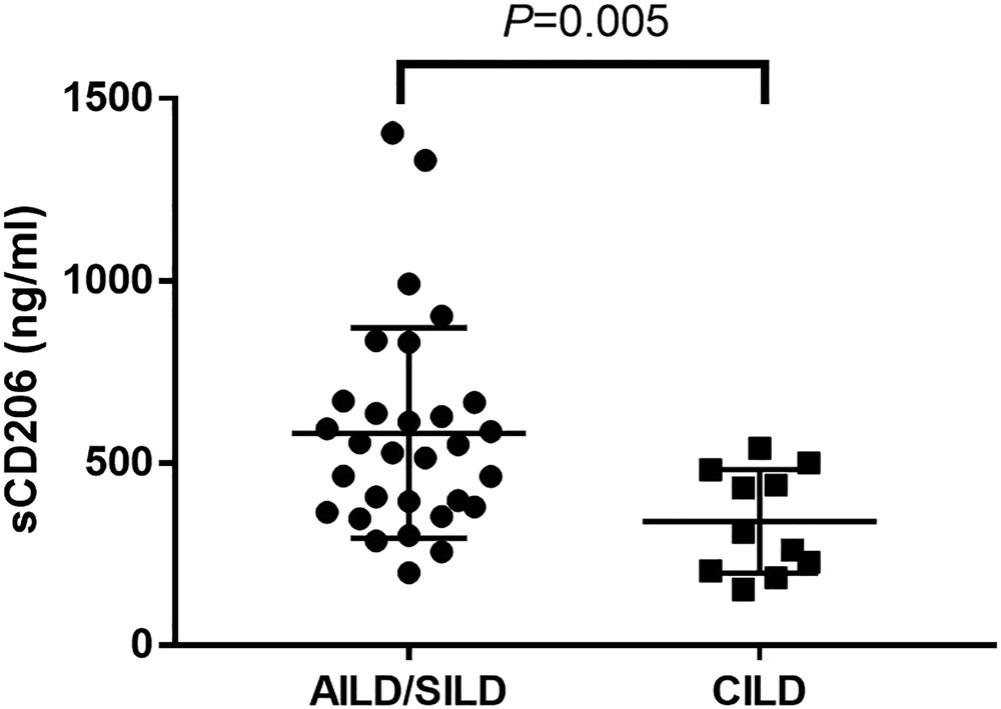
The serum soluble CD206 (sCD206) level was higher in patients with acute/subacute interstitial lung disease (AILD/SILD) (*n* = 30) than chronic interstitial lung disease (CILD) (*n* = 11).

As presented in Figure 3, the serum concentration of sCD206 was significantly higher in patients with AILD/SILD compared with CILD (539.2 ng/mL vs. 309.4 ng/mL, *P* = 0.005).

### 3.4 ROC curve analysis

**FIGURE 4:**
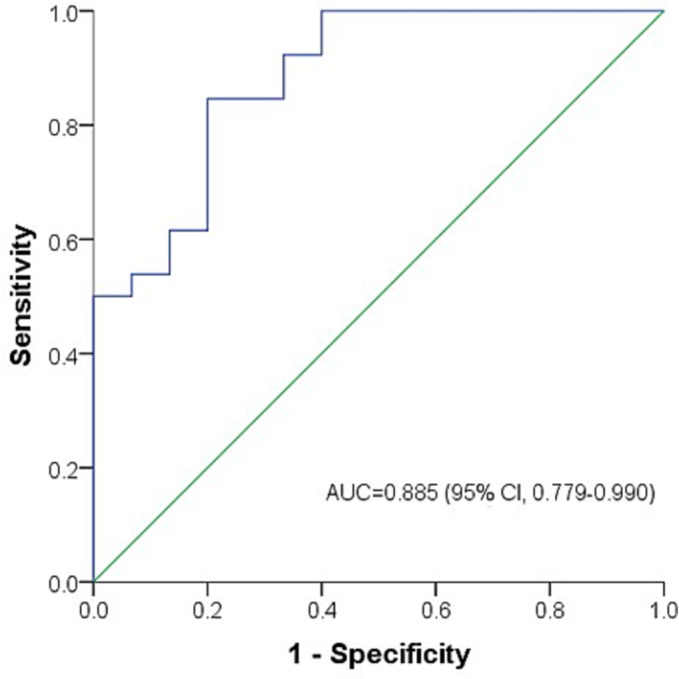
The predictive capacity of serum soluble CD206 (sCD206) levels for mortality of patients with anti‐MDA5 positive dermatomyositis/clinically amyopathic dermatomyositis‐related interstitial lung disease (MDA5‐DM/CADM‐ILD).

ROC curve was generated for serum sCD206. The area under curve (AUC) of sCD206 was 0.885 for predicting mortality (95% CI, 0.779–0.990). The optimal cut‐off value was determined as 400 ng/mL. The sensitivity was 84.6% and specificity was 80% (Figure 4).

### 3.5 Prognostic values of serum sCD206

**FIGURE 5:**
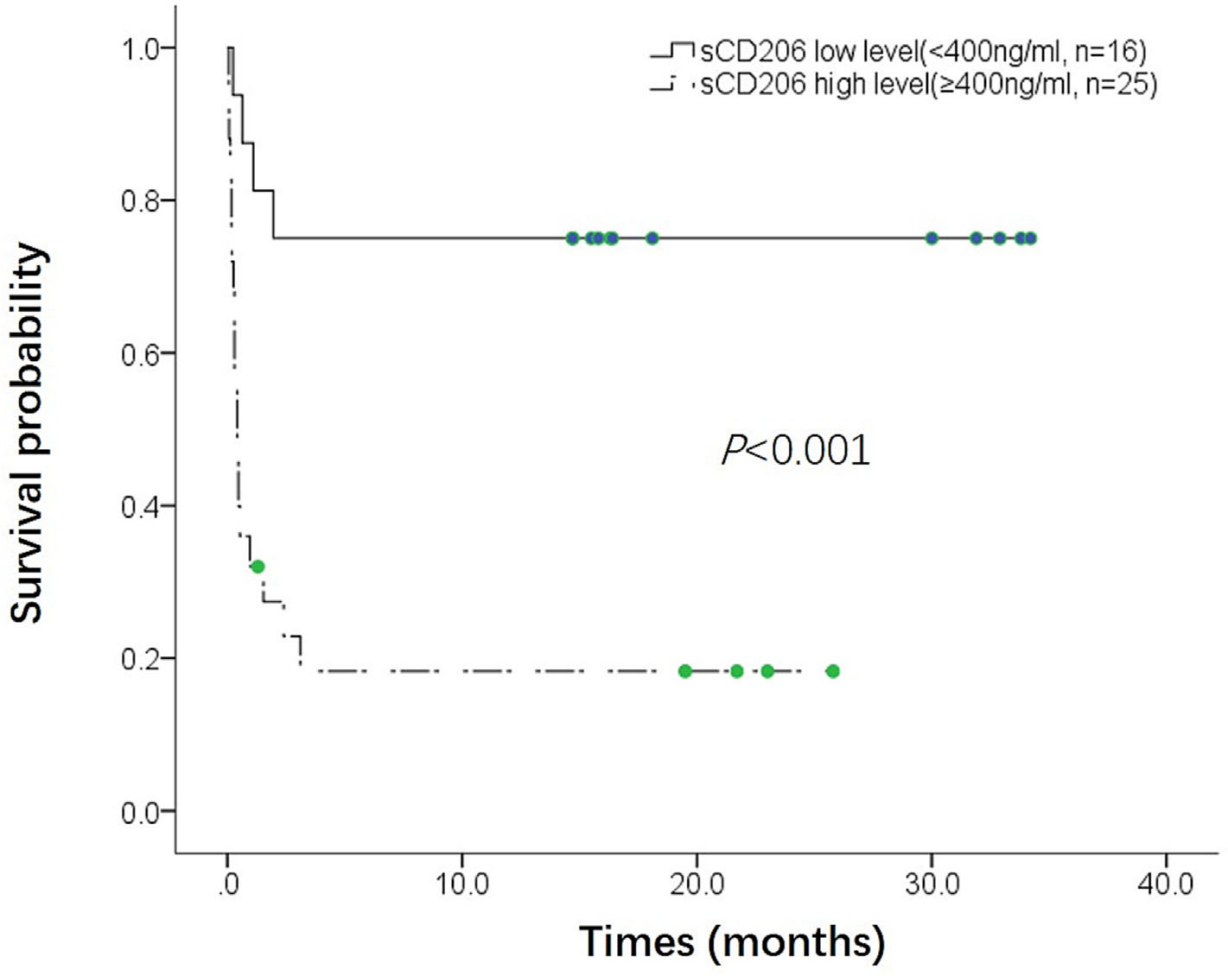
Kaplan–Meier survival curve between patients with serum sCD206 high level (≥400 ng/mL, *n* = 25) and sCD206 low level (<400 ng/mL, *n* = 16).

Using the optimal cut‐off value of sCD206, 41 patients were divided into two groups: sCD206 high level (≥400 ng/mL, *n* = 25) and low level (<400 ng/mL, *n* = 16). Kaplan–Meier curve showed the patients with sCD206 high level had significantly lower survival rate than those with sCD206 low level (25% vs. 88%, *P* < 0.001; Figure 5).

Univariate Cox hazards regression analyses were performed (Table 2). Among the baseline clinical characteristics, older age, patients with AILD/SILD, presence of fever, HRCT manifestation of DAD, elevated CRP, elevated LDH, higher serum ferritin level, and higher serum sCD206 level were all significantly associated with worse prognosis (*P*s < 0.05). While combination therapy of corticosteroids and immunosuppressants, higher PaO_2_, higher OI, higher lymphocyte count, and higher CD4+ lymphocyte count were all significantly associated with better prognosis (*P*s < 0.05). Multivariate Cox hazards regression analyses were further performed adjusting for age and gender. The significance of the following variables associated with prognosis remained: patients with AILD/SILD, presence of fever, HRCT manifestation of DAD, combination therapy of corticosteroids and immunosuppressants, PaO_2_, OI, lymphocyte count, CD4+ lymphocyte count, and sCD206. The adjusted hazard ratio of sCD206 was 1.003 (95%CI: 1.001, 1.004, *P* < 0.001). The adjusted hazard ratio for patients with sCD206 high level (≥400 ng/mL) was significantly increased compared with those with sCD206 low level (<400 ng/mL) (HR 4.857, *P* = 0.006). We further performed multivariate Cox analysis adjusting for AILD/SILD, serum ferritin, and lymphocyte count, since they were known prognostic factors for MDA5‐DM/CADM‐ILD patients. The significance of sCD206 associated with prognosis remained (HR 1.002, 95%CI: 1.001, 1.003, *P* = 0.007).

**TABLE 2:** Univariate and multivariate cox hazards analysis.

	Unadjusted HR	95% CI	*P* value	Age and gender adjusted HR	95% CI	*P* value
Age, years	1.050	1.013, 1.088	**0.008**	‐		
Female	0.678	0.313, 1.468	0.324	‐		
Former/current smokers	1.611	0.714, 3.633	0.251	1.324	0.490, 3.581	0.580
CADM	0.940	0.431, 2.047	0.875	0.771	0.347, 1.712	0.523
ILD deterioration						
CILD	Ref					
AILD/SILD	6.083	1.420, 26.056	**0.015**	5.047	1.146, 22.229	**0.032**
Fever	2.848	1.251, 6.485	**0.013**	2.871	1.254, 6.572	**0.013**
HRCT findings						
Non‐DAD	Ref			Ref		
DAD	5.456	2.318, 12.843	**<0.001**	6.356	2.393, 16.880	**<0.001**
Treatment prior to admission						
None	Ref					
CS alone	0.977	0.445, 2.143	0.953	0.822	0.370, 1.829	0.631
CS + IM	0.271	0.035, 2.076	0.209	0.285	0.036, 2.259	0.235
Treatment after admission						
CS alone	Ref					
CS + IM	0.298	0.101, 0.882	**0.029**	0.239	0.076, 0.755	**0.015**
FVC, % predicted, (*n* = 17)	1.064	0.992, 1.141	0.085	1.054	0.976, 1.139	0.177
FEV1, % predicted, (*n* = 17)	1.045	0.990, 1.102	0.110	1.021	0.968, 1.077	0.452
TLC, % predicted, (*n* = 11)	1.037	0.944, 1.139	0.447	1.112	0.942, 1.313	0.210
DLCO, % predicted, (*n* = 14)	1.026	0.969, 1.086	0.374	1.135	0.945, 1.363	0.176
PaO_2_, torr (*n* = 37)	0.957	0.925, 0.989	**0.009**	0.942	0.907, 0.978	**0.002**
OI (*n* = 37)	0.991	0.986, 0.995	**<0.001**	0.991	0.985, 0.996	**0.001**
WBC, 10^9^/L	1.078	0.940, 1.235	0.283	1.112	0.962, 1.286	0.152
PLT, 10^9^/L	0.994	0.987, 1.000	0.066	0.996	0.989, 1.004	0.327
Lymphocyte count, per 100/mm^3^ increase	0.697	0.585, 0.830	**<0.001**	0.717	0.593, 0.868	**0.001**
CD4 count, /mm^3^ (*n* = 36)	0.996	0.993, 0.999	**0.006**	0.997	0.994, 0.9998	**0.037**
NK count, /mm^3^ (*n* = 36)	1.00	0.998, 1.003	0.873	1.001	0.998, 1.003	0.521
CRP, mg/L (*n* = 38)	1.020	1.001, 1.039	**0.042**	1.018	1.000, 1.036	0.054
ESR, mm/h (*n* = 33)	0.990	0.971, 1.009	0.310	0.993	0.973, 1.014	0.523
CK, U/L (*n* = 37)	1.001	0.997, 1.005	0.704	0.999	0.994, 1.004	0.757
LDH, U/L (*n* = 38)	1.0002	1.000047, 1.000353	**0.010**	1.000	1.000, 1.000	0.085
Ferritin, ng/mL	1.00007	1.00002, 1.00012	**0.005**	1.000	1.000, 1.000	0.214
sCD206, ng/mL	1.003	1.002, 1.004	**<0.001**	1.003	1.001, 1.004	**<0.001**
<400	Ref					
≥400	6.486	2.201, 19.115	**0.001**	4.857	1.557, 15.151	**0.006**

*Note*: Bold values: *P* < 0.05.

Abbreviations: AILD/SILD, acute/subacute interstitial lung disease; CADM, clinically amyopathic dermatomyositis; CILD, chronic interstitial lung disease; CK, creatine kinase; CRP, C‐reactive protein; CS, corticosteroids; DAD, diffuse alveolar damage; DLCO, diffusing capacity of the lung carbon monoxide; DM, dermatomyositis; ESR, erythrocyte sedimentation rate; FEV1, forced expiratory volume in 1 s; FVC, forced vital capacity; HRCT, high‐resolution computed tomography; IM, immunosuppressants; LDH, lactate dehydrogenase; NK, natural killer; NSIP, nonspecific interstitial pneumonia; OI, oxygen index; OP, organizing pneumonia; PLT, platelet; TLC, total lung capacity; WBC, white blood count.

## 4 DISCUSSION

This study assessed the clinical significance of the alternative macrophage marker sCD206 in patients with MDA5‐DM/CADM‐ILD. The serum sCD206 level was elevated in patients with MDA5‐DM/CADM‐ILD and higher in patients with acute/subacute interstitial lung disease. Besides, our study suggested that serum sCD206 could predict prognosis of these patients, with elevated sCD206 associated with worse prognosis.

Mechanisms for serum sCD206 elevation in MDA5‐DM/CADM‐ILD has remained unclear. The pathophysiologic process of MDA5‐DM/CADM‐ILD included proinflammatory cytokines overproduction and alveolar macrophages activation in the lungs.^17^ Alveolar macrophages in this condition mainly show the characteristics of M2 type.^18^ M2 macrophages are characterized as immunosuppressive cells, preventing excessive inflammation and regulating tissue repair.^19^, ^20^ Activated M2 alveolar macrophages express substantial CD206 on cell surface. Immunohistochemical staining in autopsy specimens has shown alveolar macrophages positive for CD206.^21^ This membrane form of CD206 can be cleaved and released into peripheral circulation as sCD206. Elevated serum sCD206 might represent activation of M2 alveolar macrophages in MDA5‐DM/CADM‐ILD, which was in accordance with the disease pathophysiology.

In this study, serum sCD206 level showed negatively correlated with lymphocyte count and CD4+ lymphocyte count in MDA5‐DM/CADM‐ILD patients. The negative correlation between sCD206 and CD4+ lymphocyte count was also found during human immunodeficiency virus infection.^10^ Peripheral lymphopenia is common in autoimmune disease.^22^ Reduction of lymphocyte‐mediated defense function might cause macrophage activation. The mechanism underlining the correlation of lymphopenia and sCD206 elevation needs to be further clarified.

HRCT evaluation has been demonstrated useful in predicting the presence of anti‐MDA5 antibody in DM‐ILD^23^; however, the prognostic value of HRCT manifestations for MDA5‐DM/CADM‐ILD has not been fully evaluated. Our study showed that DAD manifestation in chest HRCT was significantly associated with worse prognosis. Serum sCD206 was significant higher in patients with DAD manifestation than those without DAD presence (data not shown). The severe damage caused by DAD may trigger the activation of anti‐inflammatory alveolar macrophages, leading to more CD206 being released into the peripheral circulation. Meanwhile, our study showed combination treatment of corticosteroids and immunosuppressants was significantly associated with lower risk of death. The immunosuppressants in this study included cyclophosphamide, cyclosporin A, and tacrolimus. Combination therapy of corticosteroids and immunosuppressants has been widely used to treat MDA5‐DM/CADM‐ILD. Our findings provide useful information for clinical practice regarding these patients.

Serum sCD206 levels were also found significantly correlated with serum ferritin in patients with MDA5‐DM/CADM‐ILD. CD206 and ferritin are all mainly produced by activated alveolar macrophages. However, only sCD206 proved to be an independent prognostic predictor in this study. In addition, the AUC of sCD206 was 0.885. Serum sCD206 might be a better prognostic predictor than ferritin for patients with MDA5‐DM/CADM‐ILD.

The limitation of this study is that this is a single‐center, small‐sample retrospective study, which is common in the studies regarding MDA5‐DM/CADM‐ILD, since the incidence of this unique disease is very low. Horiike et al previously reported increased sCD206 was associated with worse prognosis in MDA5‐CADM/DM‐ILD, which was consistent with our result.^24^ Our study had a bigger sample size about Chinese patients and the relationships between sCD206 and various clinical characteristics were also determined.

## 5 CONCLUSIONS

The prognosis of MDA5‐DM/CADM‐ILD patients is poor. Our study showed sCD206 was elevated in patients with acute/subacute interstitial lung disease compared with chronic interstitial lung disease, and higher serum sCD206 level was associated with worse prognosis. Serum sCD206 could be a useful noninvasive biomarker to imply ILD deterioration and predict the prognosis for MDA5‐DM/CADM‐ILD patients.

## AUTHOR CONTRIBUTIONS

Yan Li and Xiaoqin Liu contributed to collecting data, analyzing data, and writing the paper. Min Cao and Hourong Cai contributed to designing the study, interpreting data, and revising the paper. Mi Tian, Ruyi Zou, and Yujuan Gao measured serum biomarkers. Mi Tian and Mei Huang contributed to collecting data and interpreting data. Kefeng Zhou and Hourong Cai contributed to HRCT evaluation.

## CONFLICT OF INTEREST STATEMENT

All authors declare that they have no conflict of interests regarding this paper.

## ETHICS STATEMENT

This study adheres to the amended Declaration of Helsinki criteria. The research protocol was approved by the Ethics Committee of Nanjing Drum Tower Hospital. Regarding serum conservation, written informed consent was obtained from each subject. Regarding the data analyses, informed consent was waived since this study was analyzed retrospectively using anonymized clinical data.

## Data Availability

Data and materials related to this paper are available from the corresponding authors on reasonable requests.

## References

[1] Bohan A , Peter JB . Polymyositis and dermatomyositis (first of two parts). N Engl J Med. 1975;292(7):344‐347. doi:10.1056/NEJM197502132920706 1090839

[2] Fujisawa T , Hozumi H , Kono M , et al. Predictive factors for long‐term outcome in polymyositis/dermatomyositis‐associated interstitial lung diseases. Respir Investig. 2017;55:130‐137. 10.1016/j.resinv.2016.09.006 28274528

[3] Suda T , Fujisawa T , Enomoto N , et al. Interstitial lung diseases associated with amyopathic dermatomyositis. Eur Respir J. 2006;28(5):1005‐1012. doi:10.1183/09031936.06.00038806 16837503

[4] Kishaba T , McGill R , Nei Y , et al. Clinical characteristics of dermatomyosits/polymyositis associated interstitial lung disease according to the autoantibody. J Med Invest. 2018;65(3.4):251‐257. doi:10.2152/jmi.65.251 30282869

[5] Moghadam‐Kia S , Oddis CV , Sato S , Kuwana M , Aggarwal R . Anti‐melanoma differentiation‐associated gene 5 is associated with rapidly progressive lung disease and poor survival in US patients with amyopathic and myopathic dermatomyositis. Arthritis Care Res (Hoboken). 2016;68(5):689‐694. doi:10.1002/acr.22728 26414240 PMC4864500

[6] Ricardo SD , van Goor H , Eddy AA . Macrophage diversity in renal injury and repair. J Clin Invest. 2008;118(11):3522‐3530. doi:10.1172/JCI36150 18982158 PMC2575702

[7] Anders HJ , Ryu M . Renal microenvironments and macrophage phenotypes determine progression or resolution of renal inflammation and fibrosis. Kidney Int. 2011;80(9):915‐925. doi:10.1038/ki.2011.217 21814171

[8] Su Y , Bakker T , Harris J , et al. Glycosylation influences the lectin activities of the macrophage mannose receptor. J Biol Chem. 2005;280(38):32811‐32820. doi:10.1074/jbc.M503457200 15983039

[9] Suzuki Y , Shirai M , Asada K , et al. Macrophage mannose receptor, CD206, predict prognosis in patients with pulmonary tuberculosis. Sci Rep. 2018;8(1):13129. doi:10.1038/s41598-018-31565-5 30177769 PMC6120933

[10] Andersen MN , Hønge BL , Jespersen S , et al. Soluble macrophage mannose receptor (sCD206/sMR) as a biomarker in human immunodeficiency virus infection. J Infect Dis. 2018;218(8):1291‐1295. doi:10.1093/infdis/jiy318 29800140

[11] Euwer RL , Sontheimer RD . Dermatologic aspects of myositis. Curr Opin Rheumatol. 1994;6(6):583‐589. doi:10.1097/00002281-199411000-00006 7865377

[12] Sontheimer RD . Would a new name hasten the acceptance of amyopathic dermatomyositis (dermatomyositis siné myositis) as a distinctive subset within the idiopathic inflammatory dermatomyopathies spectrum of clinical illness? J am Acad Dermatol. 2002;46(4):626‐636. doi:10.1067/mjd.2002.120621 11907524

[13] Hirakata M , Nagai S . Interstitial lung disease in polymyositis and dermatomyositis. Curr Opin Rheumatol. 2000;12(6):501‐508. doi:10.1097/00002281-200011000-00005 11092199

[14] Gono T , Sato S , Kawaguchi Y , et al. Anti‐MDA5 antibody, ferritin and IL‐18 are useful for the evaluation of response to treatment in interstitial lung disease with anti‐MDA5 antibody‐positive dermatomyositis. Rheumatology (Oxford). 2012;51(9):1563‐1570. doi:10.1093/rheumatology/kes102 22589330

[15] American Thoracic Society; European Respiratory Society , American Thoracic Society/European Respiratory Society International Multidisciplinary Consensus Classification of the Idiopathic Interstitial Pneumonias . This joint statement of the American Thoracic Society (ATS), and the European Respiratory Society (ERS) was adopted by the ATS board of directors, June 2001 and by the ERS Executive Committee, June 2001. Am J Respir Crit Care Med. 2002;165:277‐304. 11790668 10.1164/ajrccm.165.2.ats01

[16] Gao Y , Zhao Q , Xie M , et al. Prognostic evaluation of serum osteopontin in patients with anti‐MDA5 antibody‐positive dermatomyositis associated interstitial lung disease. Cytokine. 2020;135:155209. doi:10.1016/j.cyto.2020.155209 32738770

[17] Fujisawa T , Hozumi H , Yasui H , et al. Clinical significance of serum chitotriosidase level in anti‐MDA5 antibody‐positive dermatomyositis‐associated interstitial lung disease. J Rheumatol. 2019;46(8):935‐942. doi:10.3899/jrheum.180825 31092718

[18] Thepen T , Van Rooijen N , Kraal G . Alveolar macrophage elimination in vivo is associated with an increase in pulmonary immune response in mice. J Exp Med. 1989;170(2):499‐509. doi:10.1084/jem.170.2.499 2526847 PMC2189410

[19] Hussell T , Bell TJ . Alveolar macrophages: plasticity in a tissue‐specific context. Nat Rev Immunol. 2014;14(2):81‐93. doi:10.1038/nri3600 24445666

[20] Gordon S , Martinez FO . Alternative activation of macrophages: mechanism and functions. Immunity. 2010;32(5):593‐604. doi:10.1016/j.immuni.2010.05.007 20510870

[21] Kaku Y , Imaoka H , Morimatsu Y , et al. Overexpression of CD163, CD204 and CD206 on alveolar macrophages in the lungs of patients with severe chronic obstructive pulmonary disease. PLoS ONE. 2014;9(1):e87400. doi:10.1371/journal.pone.0087400 24498098 PMC3907529

[22] Sowden E , Carmichael AJ . Autoimmune inflammatory disorders, systemic corticosteroids and pneumocystis pneumonia: a strategy for prevention. BMC Infect Dis. 2004;4(1):42. doi:10.1186/1471-2334-4-42 15488151 PMC526257

[23] Hozumi H , Fujisawa T , Nakashima R , et al. Comprehensive assessment of myositis‐specific autoantibodies in polymyositis/dermatomyositis‐associated interstitial lung disease. Respir Med. 2016;121:91‐99. doi:10.1016/j.rmed.2016.10.019 27888997

[24] Horiike Y , Suzuki Y , Fujisawa T , et al. Successful classification of macrophage‐mannose receptor CD206 in severity of anti‐MDA5 antibody positive dermatomyositis associated ILD. Rheumatology (Oxford). 2019;58(12):2143‐2152. doi:10.1093/rheumatology/kez185 31143953

